# Permanganate of Potassa in the Treatment of Certain Female Diseases

**Published:** 1871-01

**Authors:** A. M. Williams

**Affiliations:** Springboro, Crawford County, Pa.


### PERMANGANATE OF POTASSA IN THE TREATMENT OF CERTAIN FEMALE DISEASES.

By A. M. Williams, A. M., M. D., of Springboro, Crawford County, Pa.

Dr. Williams desires to call attention to certain uses of permanganate of potash, which, so far as he is aware, have not been alluded to by writers. This salt has of late received favorable notice in the journals, and is certainly one of the most valuable articles of the Materia Medica to the surgeon. From his own experience Dr. Williams is led to believe that it may likewise be made of great value to the obstetrician and gynæcologist. The cases in which the author has found this salt most useful have been those of *excessive purulent* and *prolonged lochial discharge.*

A year ago Dr. Williams was called to see a woman who had been confined about two weeks previous to the visit. She was a primipara, small and of strumous constitution. The person who attended her during labor was a homœpathist. The labor seems to have been an easy one; the lochia was more than usually abundant, and on the fifth or sixth day it became purulent and highly offensive, growing more so continuously until the author was called. The patient and her husband became much alarmed, and sent for the doctor who had attended her, but were assured that nothing could be done; that it would cease in time, and that interference would be positively dangerous. On Dr. Williams's first visit the discharges were so indescribably offensive that no one could long endure to remain in the room. With a view chiefly to remove this, and for purposes of cleanliness, a solution of permanganate of potash was ordered, grains 10 in a pint of tepid water, to be used by injection, copiously, twice a day. In two days Dr. Williams made another visit, found all the stench removed, and the discharges not only natural in appearance, but far less copious. The treatment was continued two days longer, and the patient was pronounced cured.

The happy manner in which the salt acted in this case induced Dr. Williams to try it further in similar cases, and always with like results. He had used it, however, with a view only of destroying the fetor, but he observed not only a speedy change in the character of the discharge, but a marked diminution in the *quantity*. This led him to presume that in simple cases of *excessive lochia* it might be of service. Dr. Williams regrets that he has not had more abundant opportunities for testing this, and will only say that in two cases of prolonged and excessive discharge that have recently come under his notice, the use of this agent was followed by very speedy relief.—*Half Yearly Abstract.*

